# Wedelolactone Regulates Lipid Metabolism and Improves Hepatic Steatosis Partly by AMPK Activation and Up-Regulation of Expression of PPARα/LPL and LDLR

**DOI:** 10.1371/journal.pone.0132720

**Published:** 2015-07-13

**Authors:** Yun Zhao, Lu Peng, Li-chao Yang, Xu-dong Xu, Wei-jie Li, Xiu-mei Luo, Xin Jin

**Affiliations:** 1 Department of Basic Medical Sciences of Medical College, Xiamen University, Xiamen, Fujian, China; 2 Xiamen Key Laboratory of Chiral Drugs, Xiamen, Fujian, China; 3 Department of Cardiology, Zhongshan Hospital, Xiamen, Fujian, China; Northeast Ohio Medical University, UNITED STATES

## Abstract

Hyperlipidemia is considered one of the greatest risk factors of cardiovascular diseases. We investigated the anti-hyperlipidemic effect and the underlying mechanism of wedelolactone, a plant-derived coumestan, in HepG2 cells and high-fat diet (HFD)−induced hyperlipidemic hamsters. We showed that in cultured HepG2 cells, wedelolactone up-regulated protein levels of adenosine monophosphate activated protein kinase (AMPK) and peroxisome proliferator-activated receptor-alpha (PPARα) as well as the gene expression of AMPK, PPARα, lipoprotein lipase (LPL), and the low-density lipoprotein receptor (LDLR). Meanwhile, administration of wedelolactone for 4 weeks decreased the lipid profiles of plasma and liver in HFD−induced hyperlipidemic hamsters, including total cholesterol (TC), triglycerides (TG), and low-density lipoprotein-cholesterol (LDL-C). The activation of AMPK and up-regulation of PPARα was also observed with wedelolactone treatment. Furthermore, wedelolactone also increased the activities of superoxidase dismutase (SOD) and glutathione peroxidase (GSH-Px) and decreased the level of the lipid peroxidation product malondialdehyde (MDA) in the liver, therefore decreasing the activity of alanine aminotransferase (ALT). In conclusion, we provide novel experimental evidence that wedelolactone possesses lipid-lowering and steatosis-improving effects, and the underlying mechanism is, at least in part, mediated by the activation of AMPK and the up-regulation of PPARα/LPL and LDLR.

## Introduction

Hyperlipidemia is a metabolic disorder characterized by increased levels of TC, LDL-C, very low-density lipoprotein-cholesterol (VLDL-C), and TG, with a concomitant decrease in the level of high-density lipoprotein-cholesterol (HDL-C) circulating in the blood. High serum levels of lipids are a risk factor for the development of cardiovascular diseases, including atherosclerosis, coronary heart disease, and hypertension. Meanwhile, lipid accumulation in the liver (steatosis) results in inflammation and oxidative stress, leading to liver damage [1,2].

Wedelolactone is a plant-derived coumarin. It is an important active small-molecule compound with many pharmacological activities including anti-fibrotic effects on the human hepatic stellate cell line LX-2 [3], inhibitory effects on the proliferation and differentiation of pre-osteoclasts [4], and anti-HCV activity [5]. The lipid-lowering effects of wedelolactone have not been reported. However, *Eclipta prostrate L*, which is a traditional Chinese herbal medicine whose characteristic compound is wedelolactone, has been demonstrated to have hypolipidemic activity [6]. This current study is the first to determine the hypolipidemic effects of wedelolactone and investigate its underlying mechanism.

Interestingly, wedelolactone has been found to be a potent and selective inhibitor of I kappa B kinases [7] and 5-lipoxygenase [8], making it a candidate drug for the prevention and treatment of inflammation. Inflammation is associated with disturbance of reactive oxygen species (ROS) within the pathogenesis of hyperlipidemia and contributes to liver damage. Therefore, hyperlipidemia is usually accompanied by liver damage; theoretically, lipid-lowering process by drugs can improve liver damage. However, a few studies reveal that several anti-hyperlipidemia drugs may induce liver damage [9,10]. Therefore, we also explored the beneficial effects of wedelolactone in reducing liver damage as well as its lipid-regulating effect.

## Materials and Methods

### Ethical approval of the study protocol

All animal experimental procedures were in accordance with the *Guide for the Care and Use of Laboratory Animals* (8th edition; US National Institutes of Health Publication, Bethesda, MD, USA, 2011) and the protocol was approved by the Animal Care and Use Committee of Xiamen University (Xiamen, China).

### Procedures of cell culture

HepG2 cells were cultured in Dulbecco’s modified Eagle’s medium (DMEM)/high glucose (HyClone; Thermo Scientific, Jülich, Germany) with 10% fetal bovine serum (FBS) (Gemini Bio Products, Sacramento, CA, USA) at 37°C with 5% CO_2_ until ≈70% confluent. Cells were synchronized in DMEM containing 0.4% FBS for 24 h before treatment. To inhibit the activation of PPARα, cells were pre-treated with MK886 for 0.5 h, followed by treatment of wedelolactone (Jingke Chemical Science and Technology, Shanghai, China) for 24 h.

### Cell viability assay

The MTS assay was employed to evaluate the toxicity of wedelolactone on the human liver hepatocellular carcinoma cell line HepG2 (Cell Resource Center of the Shanghai Institutes for Biological Sciences, Chinese Academy of Sciences, Shanghai, China). Briefly, HepG2 cells were plated at 2×10^3^ cells per well in 96-well plates in DMEM containing 10% FBS at 37°C. After overnight incubation, cells were synchronized in DMEM containing 0.4% FBS for 24 h and stimulated with various concentrations of wedelolactone for a further 24 h. Cell viability was measured by a CellTiter 96 AQueous One Solution Cell Proliferation Assay kit (Promega, Fitchburg, WI, USA). Viability was calculated using the following formula:

Viability (%)=A490(sample)/ A490(control)×100%

Absorbance was measured at 490 nm on a Microplate Reader (Molecular Devices, Silicon Valley, CA, USA).

### Quantitative real-time polymerase chain reaction (qPCR)

Relative mRNA expression of PPARα, LPL, LDLR, AMPK, carnitine palmitoyltransferase 1 (CPT1), cluster of differentiation 36 (CD36), acetyl-CoA carboxylase (ACC), sterol regulatory element binding protein-1c (SREBP-1c), sterol regulatory element binding protein-2 (SREBP-2), and SREBPs’ downstream genes, including hydroxy-3-methyl-glutaryl-CoA reductase (HMGCR), fatty acid synthetase (FAS) and stearoyl-CoA desaturase (SCD) [11] in HepG2 cells were determined by qPCR.

Total cellular RNA was extracted using TRIzol (Invitrogen, Carlsbad, CA, USA) following the manufacturer’s instructions. cDNA synthesis was undertaken using a Reverse Transcription kit (Thermo Fisher, Boston, MA, USA). Primers were synthesized by Sangon Biotechnology (Shanghai, China) and are listed in Table 1.

**Table 1:** Primer sequences used for qPCR.

Genes	Forward primer (5'-3')	Reverse primer(5'-3')
PPARα	TCCTGAGCCATGCAGAATTTAC	AGTCTAAGGCCTCGCTGGTG
LPL	GCTCAGGAGCATTACCCAGTGTC	GCTCCAAGGCTGTATCCCAAGA
LDLR	TTGCAAACCCTGGTTGCTGTA	CACAGTCCAGTTCGTGCAAATAATC
AMPK	GTCATGATAGCTTGCATAAATGGTG	AGTTGAATAGAACAAGCCCTGGAC
GAPDH	GCACCGTCAAGGCTGAGAAC	TGGTGAAGACGCCAGTGGA
SREBP1c	CAGCCCCACTTCATCAAGG	ACTGTTGCCAAGATGGTTCCG
SREBP2	AACGGTCATTCACCCAGGTC	GGCTGAAGAATAGGAGTTGCC
ACC	TCCGCACTGACTGTAACCAC	GCGACTTCCATACCGCATTA
HMGCR	AAGGAGGCATTTGACAGCAC	CTGACCTGGACTGGAAACG
FAS	TGTGGACATGGTCACGGAC	GGCATCAAACCTAGACAGGTC
CD36	AATGTAACCCAGGACGCTGA	AGCCAGATTGAGAACTGTGAAG
SCD	TTCCCGACGTGGCTTTTTCT	AGCCAGGTTTGTAGTACCTCC
CPT1	GGTGGAACAGAGGCTGAAGT	AAAGGCAGAAGAGGTGACGA
GAPDH	GCACCGTCAAGGCTGAGAAC	TGGTGAAGACGCCAGTGGA

qPCR was performed with the 7300 Real Time PCR System (Applied Biosystems, Life Technologies Corporation, CA, USA). Gene expression was measured using SYBR Green (Applied Biosystems). The cycling program was set as follows: thermal activation for 30 s at 95°C and 40 cycles of PCR (melting for 5 s at 95°C, followed by annealing/extension for 31 s at 60°C). PPARα, LPL, LDLR, AMPK, CPT1, CD36, ACC, SREBP-1c, SREBP-2, HMGCR, FAS and SCD gene expression data for individual samples were normalized to the corresponding control glyceraldehyde-3-phosphate dehydrogenase (GAPDH) gene expression.

### Western blot analyses

Protein was extracted using RIPA-reagent LS (Aidlab Biotechnologies, Beijing, China) according to the manufacturer’s protocols. Protein level was measured using a Bicinchoninic Acid kit (Thermo Scientific). Then, 40 μg of protein lysate was separated by 10% sodium dodecyl sulfate–polyacrylamide gel electrophoresis, transferred onto polyvinylidene difluoride membranes (Millipore, Bedford, MA, USA), and then immunoblotted with antibodies against phospho-AMPK_α1_ (Thr-172; 1:1000 dilution; Cell Signaling Technology, Danvers, MA, USA), AMPK (1:1000; Abcam, Cambridge, UK), PPARα (1:1000; Abcam), and GAPDH (1:2000; Abcam). Subsequently, they were incubated with horseradish peroxidase-conjugated secondary antibodies (MultiSciences (Lianke) Biotech, Hangzhou, China), visualized using an ECL Plus kit (Millipore) and exposed by autoradiography (Eastman Kodak, Rochester, NY, USA).

### Antioxidant activity of wedelolactone *in vitro*

The antioxidant activity of wedelolactone *in vitro* was measured using 2,2-diphenyl-1-picrylhydrazyl (DPPH) and SOD activity assays.

The DPPH radical-scavenging activities of wedelolactone were examined in comparison with ascorbic acid, a known antioxidant, (Sinopharm Chemical Reagents, Shanghai, China) using a method previously reported [12]. Briefly, various concentrations of samples (1.0, 0.5, 0.125, 0.1, 0.08, 0.0625, 0.05, 0.03125 mg/mL) were mixed with 3 mL of an ethanol solution of DPPH (0.04 g/L; Sigma-Aldrich, St. Louis, MO, USA). The mixture was shaken vigorously and allowed to stand at room temperature for 0.5 h. Absorbance was measured at 517 nm in a SpectraMax M2 Microplate Reader (Molecular Devices, Silicon Valley, CA, USA). Lower absorbance of the reaction mixture indicated a higher activity of scavenging of free radicals. The percentage of DPPH discolored samples was calculated according to the following formula: Anti-radical activity (%) = [A_1_ –(A_S_ - A_0_)]/A_1_×100%. A_0_ is the blank control absorbance value (containing anhydrous ethanol) used to eliminate the color effect of the sample itself; A_1_ serves as the control absorbance value (containing only the DPPH ethanol solution); A_S_ is the absorbance value of wedelolactone at different concentrations.

SOD activity in the cell study was measured by the xanthine oxidase method using a detection kit from the Nanjing Jiancheng Bioengineering Institute. Briefly, HepG2 cells were plated at 2×10^5^ cells per well in 6-well plates in DMEM containing 10% FBS at 37°C. After overnight incubation, cells were synchronized in DMEM containing 0.4% FBS for 24 h and stimulated with 25 μM oleic acid (OA) or OA with various concentrations of wedelolactone for an additional 24 h. Then, the SOD activity in HepG2 cells was measured using the detection kit.

### Animals and experimental design

Thirty-two adult KM mice weighing 24–28 g (SLAC Laboratory Animal Co. Ltd., Shanghai, China, license number: SCXK (Shanghai) 2007–0005) and sixty male Syrian golden hamsters weighing 90–100 g (Vital River Laboratory Animal Technology, Beijing, China) were housed in an environmentally controlled room with a 12 h / 12 h light / dark cycle at 22 ± 0.2°C and received a diet of standard pellets and water ad libitum for an acclimatization of 7 days before experiment.

#### Triton-induced acute hyperlipidemia

Acute hyperlipidemia was induced by intraperitoneal injection of Triton WR-1339 (Sigma-Aldrich) and used for the screening assay. KM mice were randomly divided into four groups of eight. One group served as the normal group and another as the Triton group; the remaining two groups served as test groups. The normal group underwent intraperitoneal administration of physiologic (0.9%) saline and the other groups of mice were injected with Triton WR-1339 (400 mg/kg body weight). Then, the normal group and the Triton group were treated with distilled water, whereas the test groups were treated with fenofibrate (100 mg/kg) or wedelolactone (100 mg/kg) by intragastric administration. After 16 h, mice were anesthetized with 10% chloral hydrate (4 mL/kg). Blood samples were collected through the orbital sinus and immediately centrifuged at 1123 × g for 10 min at room temperature. Sera were stored at -80°C for biochemical analyses.

#### HFD−induced hyperlipidemia

To evaluate the lipid-lowering effect of wedelolactone, an HFD-induced model of hyperlipidemia in hamsters was employed. Animals were randomly divided into six groups of 10: normal; HFD; HFD + xuezhikang (a well-used hypolipidemic Chinese patent drug); HFD + WDL10; HFD + WDL25; and HFD + WDL40. The normal group was fed a normal diet. The other groups were fed a HFD, which contained 80% normal rodent chow, 10% fat, and 2% cholesterol (Solabio, Paris, France). The latter four groups were administered xuezhikang (250 mg/kg/day; Peking University WBL Biotech, Beijing, China) *via* the intragastric route and three doses of wedelolactone (10, 25, and 40 mg/kg/day). The normal chow diet group and the HFD group were simultaneously given the same volume of vehicle.

At the end of the 4-week period, after fasting overnight, the hamsters were weighed and anesthetized with 10% chloral hydrate (4 mL/kg). Their blood samples were collected from cardiac puncture, centrifuged at 1123 × g for 10 min at room temperature, and stored at -80°C for future serum analysis. Livers were removed and stored at -80°C before use.

### Measurement of lipids and enzymes

Liver samples were homogenized (10%, *w/v*) in alcohol and then centrifuged at 1000 × *g* for 15 min at room temperature. Supernatants were used for analysis of hepatic levels of TC and TG. Supernatants from homogenates in cold saline were used for the assay of SOD, MDA, and GSH-Px.

Plasma levels of TC, TG, and LDL-C, as well as hepatic levels of TC and TG, were determined using enzymatic kits (Nanjing Jiancheng Bioengineering Institute, Nanjing, China). Samples were analyzed with a SpectraMax M2 Microplate Reader (Molecular Devices).

SOD activity was measured by the xanthine oxidase method. GSH-Px activity was determined based on an NADPH-coupled reaction. MDA content was analyzed using the thiobarbituric acid method. Activity of ALT in plasma was measured by enzymatic methods according to the manufacturer’s instructions. All detection kits were obtained from the Nanjing Jiancheng Bioengineering Institute.

### Liver histology

Livers were fixed in 4% paraformaldehyde, embedded in tissue-freezing medium (Sakura Finetek, Torrance, CA, USA), and stored at -80°C. Frozen tissue was cut into sections (thickness, 5 μm and 10 μm) and placed on slides. Tissue sections were visualized by staining with hematoxylin and eosin (H&E) and Oil-Red O.

### Statistical analyses

Results were presented as the mean ± SD. Data were analyzed by one-way ANOVA. Comparisons between two groups were made using the Student–Newman–Keuls method. A p value <0.05 was considered statistically significant. Statistical analysis and graphical presentation of data were conducted using Prism 5 (GraphPad, Avenida, CA, USA).

## Results

### Effects of wedelolactone on cell viability of HepG2 cells

We first measured the cytotoxic effect of wedelolactone in HepG2 cells. HepG2 cells were cultured until 90% confluence was reached and the cytotoxicity was assessed by the MTS assay. We observed no significant change in cell viability when cells were treated with wedelolactone at concentrations of 10, 20, 30, 40 and 50 μM. In addition, the relative growth rate (RGR) of cells (Table 2) in the presence of wedelolactone at different concentrations was more than 90% of that observed in control cells. According to the *American Pharmacopeia*, these concentrations of wedelolactone were classified as cytotoxic grade 1 and were therefore not considered to have an appreciable cytotoxicity effect on HepG2 cells.

**Table 2:** RGR of cells after treatment of wedelolactone.

Groups	Concentrations	RGR(%)
Control	-	100
DMSO	1‰	98.28±1.640
WDL10	10 μM	99.07±0.4837
WDL20	20 μM	98.99±0.7994
WDL30	30 μM	95.32±1.300*
WDL40	40 μM	94.89±2.368**
WDL50	50 μM	94.39±1.726**

The values are presented as the mean ± SD of triplicate assays.

*p<0.05 and

**p<0.01 compared to control.

### Wedelolactone regulates lipid metabolism-related genes and proteins *in vitro*

**Fig 1:**
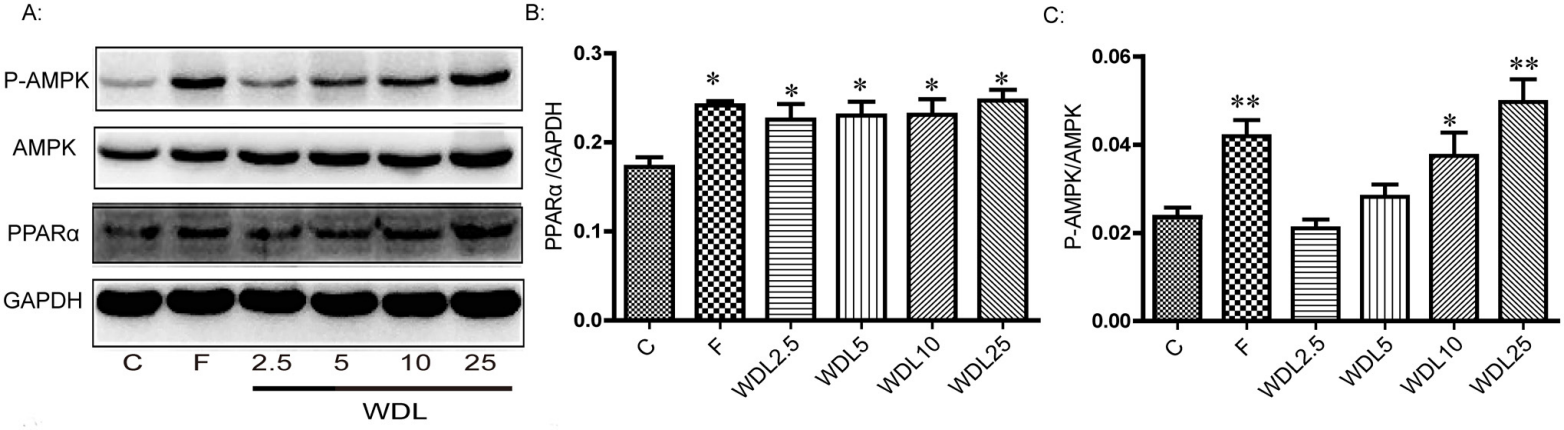
Wedelolactone activated PPARα and phospho-AMPK. (A): HepG2 cells were treated with or without wedelolactone at different concentrations for 24 h before collection of cell proteins and western blotting. (B) and (C): Protein levels were standardized against those of GAPDH. Histogram panels represent the ratios of PPARα/GAPDH (B) and phospho-AMPK/total AMPK (C). These values were obtained by quantification of the intensity of each band of the western blot. The values are expressed as the mean ± SD of triplicate assays. *p<0.05, **p<0.01 *vs*. control group.

**Fig 2:**
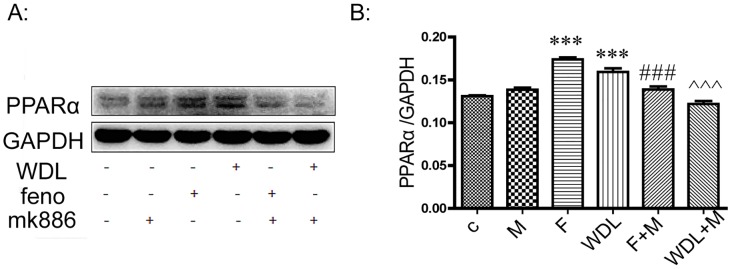
Up-regulation of PPARα expression was reversed by pre-treatment of MK886. HepG2 cells were pretreated with MK886 (15 μM) for 0.5 h before the administration of fenofibrate (100 μM) or wedelolactone (25 μM) for 24 h. Quantification of PPARα was normalized by GAPDH. The values are expressed as the mean ± SD of triplicate assays. ***p<0.001 *vs*. control, ^###^p<0.001 *vs*. fenofibrate alone, ^^^^^p<0.001 *vs*. wedelolactone alone.

We then determined by western blot whether wedelolactone affected two very important lipid-regulating proteins: PPARα and AMPK. It was shown that wedelolactone administration significantly increased the protein levels of PPARα and activated AMPK in a dose-dependent manner (Fig 1). Up-regulation of PPARα expression was reversed by pre-treatment with MK886 for 0.5 h (Fig 2).

**Fig 3:**
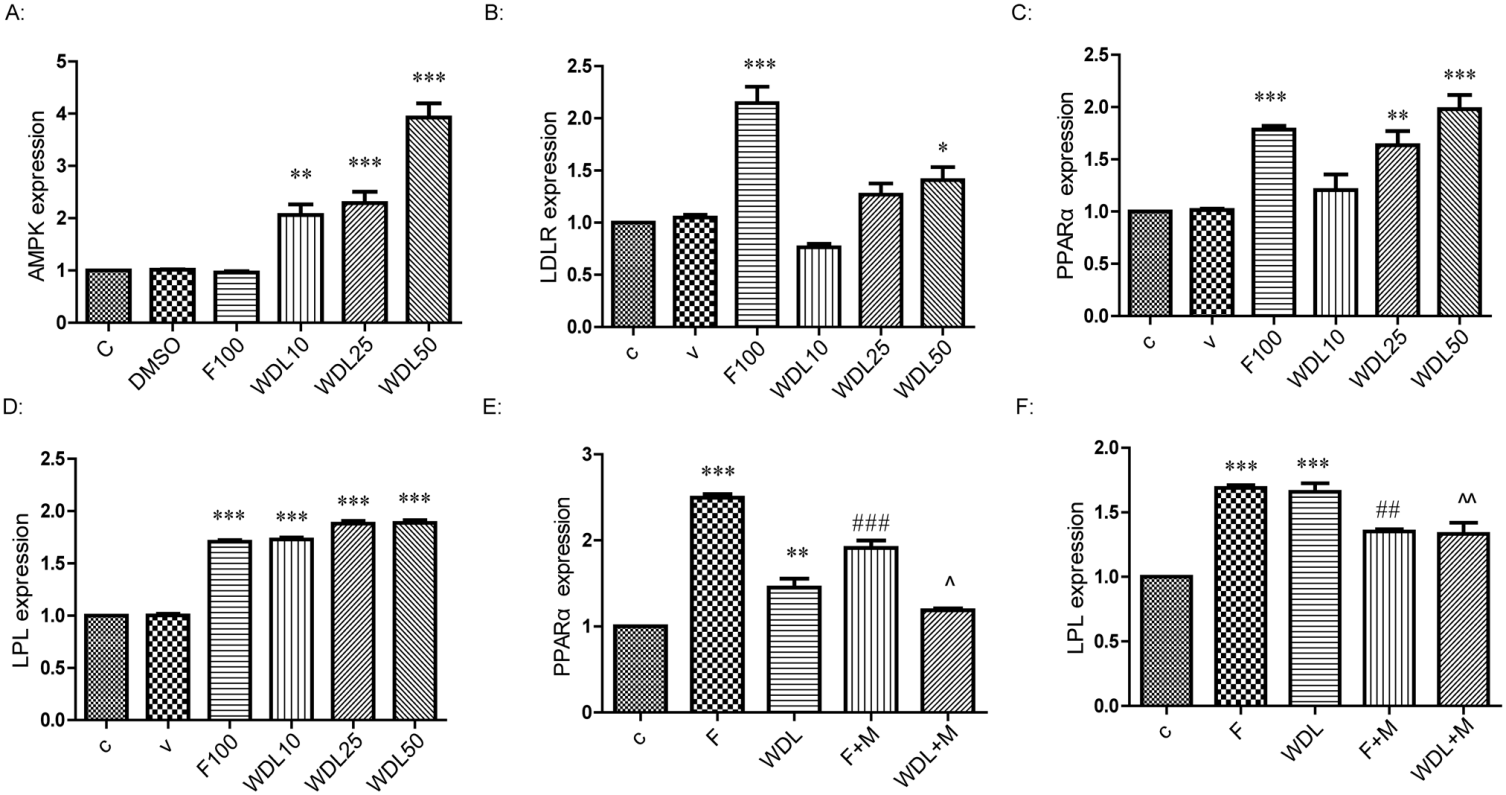
Wedelolactone altered the expression of genes related to lipid metabolism. (A), (B), (C) and (D): Effect of wedelolactone on mRNA expression in HepG2 cells treated with or without wedelolactone for 24 h. Vehicle and experimental groups were supplemented concurrently with DMSO, fenofibrate (100 μM) or wedelolactone (10 μM, 25 μM, 50 μM). (E) and (F): HepG2 cells were pretreated with MK886 (15 μM) for 0.5 h before administration of fenofibrate (100 μM) and wedelolactone (25 μM) for 24 h. The values are expressed as the mean ± SD of triplicate assays. *p<0.05, **p<0.01, ***p<0.001 *vs*. control, ^##^p<0.01, ^###^p<0.001 *vs*. fenofibrate alone, ^^^p<0.05, ^^^^p<0.01 *vs*. wedelolactone alone.

**Fig 4:**
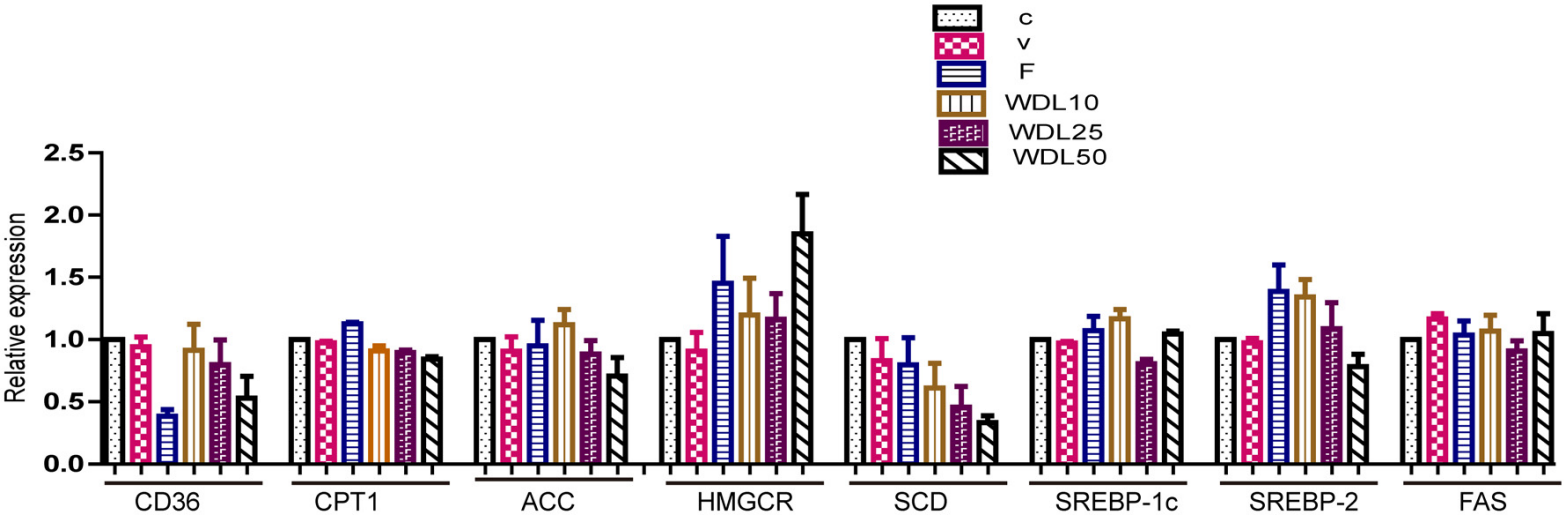
Some gene expression was not affected by wedelolactone treatment. Effect of wedelolactone on gene expressions in HepG2 cells with or without treatment of wedelolactone for 24 h. Vehicle and test groups were concurrently supplemented with DMSO, fenofibrate (100 μM) or wedelolactone (10 μM, 25 μM, 50 μM). The values are expressed as the mean ± SD of triplicate assays.

To evaluate the effects of wedelolactone on gene expression, we detected the genes involved in lipid metabolism, especially the genes involved with the pathways of PPARα and AMPK, using qPCR. The specificity of all the primers was demonstrated by both the solubility curves and the agarose gel strips of the mix of qPCR experiments (S1 and S2 Figs). It was revealed that the mRNA expressions of AMPK, LDLR, PPARα and LPL were significantly up-regulated by wedelolactone (Fig 3A–3D). Other genes we measured, such as CPT1 and CD36, were not significantly affected by wedelolactone (Fig 4).

With a peroxisome proliferator response element (PPRE) in the promoter region, LPL is reported to be a target of PPARα [13,14]. LPL expression after treatment of wedelolactone in HepG2 cells was increased dramatically at the mRNA level (Fig 3D) along with the up-regulation of PPARα both at the mRNA expression level (Fig 3C) and at the protein level (Fig 1A–1B). Up-regulation of LPL expression was reversed by pre-treatment with MK886 (PPARα antagonist) for 0.5 h (Fig 3F), along with inhibition of the mRNA level (Fig 3E) and the protein level of PPARα (Fig 2). These findings suggest that following wedelolactone treatment in HepG2 cells, LPL is a downstream target of PPARα, and that wedelolactone up-regulates LPL expression via PPARα up-regulation. Previous studies report that activation of PPARα typically induces the up-regulation of CPT1 and CD36 expression [15,16]. However, in HepG2 cells, CPT1 and CD36 remained unchanged after wedelolactone treatment in our study (Fig 4).

LDLR mediates endocytosis of cholesterol-rich LDL by hepatocytes. The mRNA level of LDLR was markedly increased by wedelolactone (Fig 3B), suggesting a potential mechanism by which wedelolactone reduces LDL-C and TC.

In addition to PPARα/LPL and LDLR, mRNA transcription of AMPK was also significantly increased by wedelolactone (Fig 3A). However, gene expression downstream of AMPK (ACC, SREBP2, SREBP-1c, and the downstream genes of SREBPs, including HMGCR, FAS, and SCD) was not significantly altered in HepG2 cells after treatment with wedelolactone (Fig 4).

These results indicated that wedelolactone up-regulated LDLR and PPARα/LPL and activated AMPK in the cultured HepG2 cells.

### Effect of wedelolactone on Triton-induced hyperlipidemia

**Fig 5:**
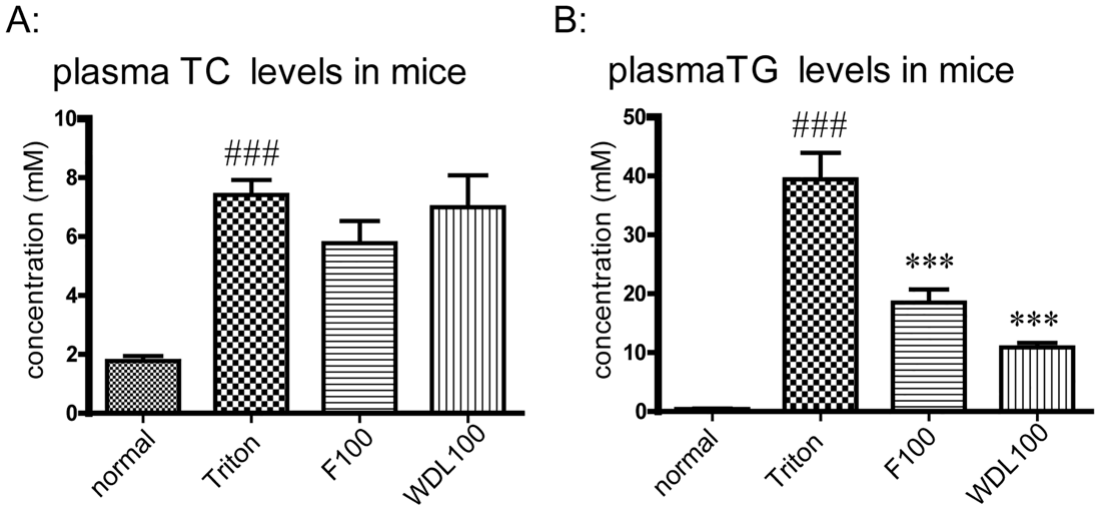
Effect of wedelolactone on plasma levels of TC and TG in mice. The normal group underwent intraperitoneal administration of physiologic (0.9%) saline and the other groups were injected with Triton WR-1339. The normal group and Triton group were treated with distilled water by intragastric administration. Test groups were treated with fenofibrate (100 mg/kg) or wedelolactone (100 mg/kg) by intragastric administration, respectively. The values are shown as the mean ± SD from eight animals in each group. ^###^p<0.001, Triton group *vs*. normal group, ***p<0.001, test group *vs*. Triton group.

Next, we illustrated the effects of wedelolactone in the *in vivo* hyperlipidemia models induced by Triton WR-1339 in mice. It was demonstrated that Triton WR-1339 dramatically elevated plasma levels of TC and TG in mice compared with the normal group; meanwhile, wedelolactone and fenofibrate significantly decreased TG levels compared with the Triton group (Fig 5B). However, neither fenofibrate nor wedelolactone showed significant effects on plasma levels of TC (Fig 5A).

### Wedelolactone alleviates liver steatosis and decreases plasma levels of TC, TG, and LDL-C in HFD hamsters

**Fig 6:**
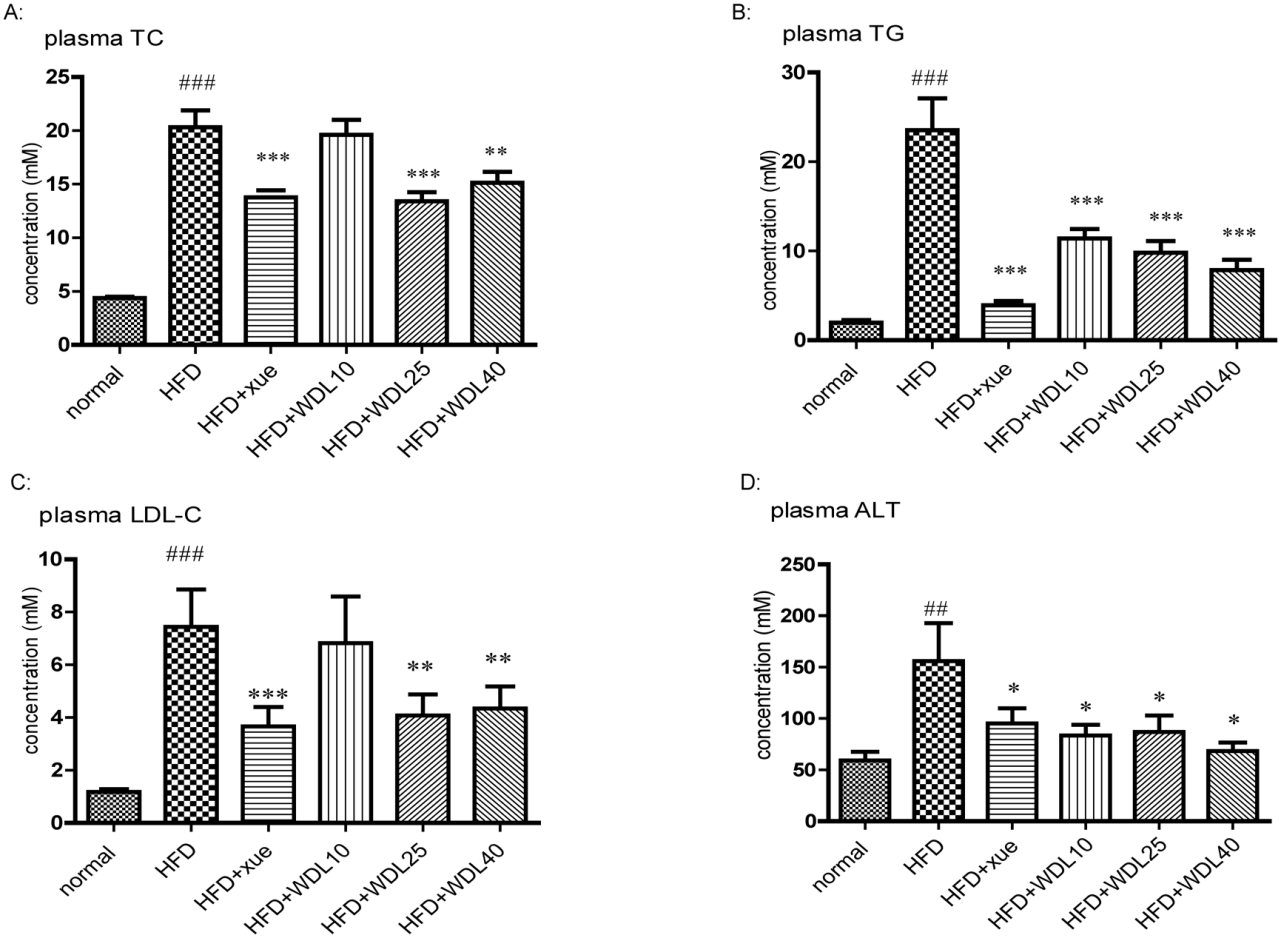
Effects of wedelolactone on plasma levels of TC (A), TG (B), LDL-C (C), and ALT (D) in Syrian hamsters. The normal group was fed a normal diet whereas the other groups were fed a HFD. HFD + xue and HFD + WDL groups were supplemented concurrently with xue (xuezhikang) (250 mg/kg) or wedelolactone (10 mg/kg, 25 mg/kg, 40 mg/kg) for 4 weeks. The values are shown as the mean ± SD from ten animals in each group. ^##^p<0.01, ^###^p<0.001 *vs*. normal group, *p<0.05, **p<0.01, ***p<0.001 *vs*. HFD group.

**Fig 7:**
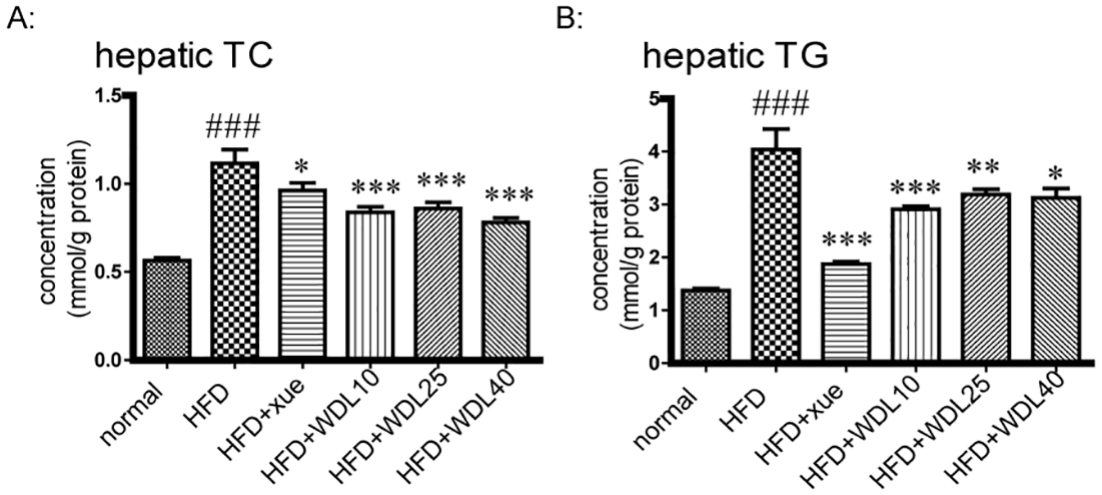
Hepatic levels of TC (A) and TG (B) in Syrian hamsters. The normal group was fed a normal diet whereas the other groups were fed a HFD. The HFD + xue and HFD + WDL groups were supplemented concurrently with xuezhikang (250 mg/kg) or wedelolactone (10 mg/kg, 25 mg/kg, 40 mg/kg). After 4 weeks, livers were homogenized and levels of TC and TG were measured. The values are shown as the mean ± SD from ten animals in each group. ^###^p<0.001 *vs*. normal group, *p<0.05, **p<0.01, ***p<0.001 *vs*. HFD group.

**Fig 8:**
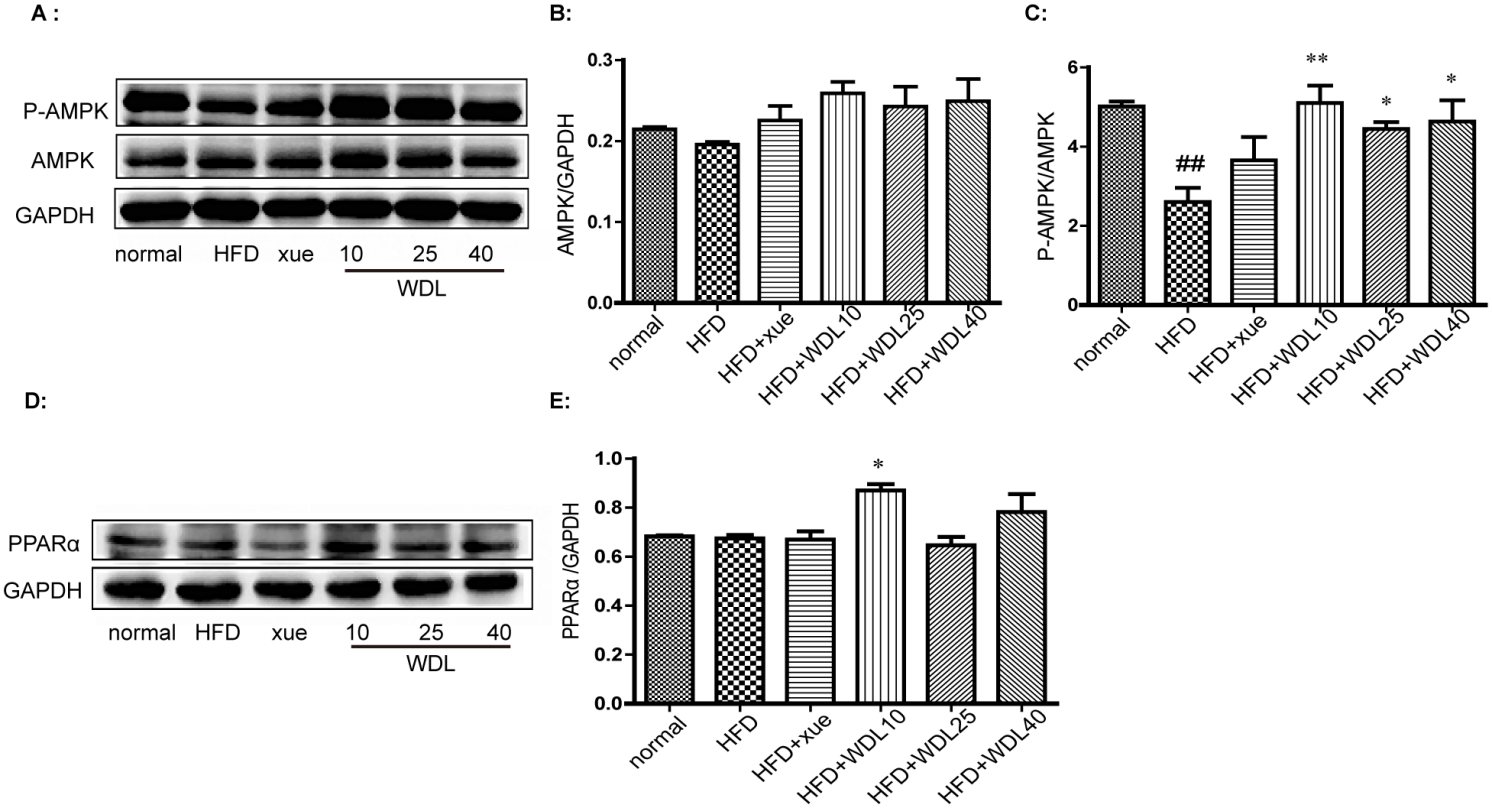
Wedelolactone activated AMPK and PPARα in Syrian hamsters. Treatment with xuezhikang (250 mg/kg) or wedelolactone (10 mg/kg, 25 mg/kg, 40 mg/kg) for four weeks significantly increased the level of phospho-AMPK (A, C) and PPARα (D, E) in liver tissues. Protein levels were standardized against GAPDH levels. The histograms represent the ratios of AMPK/GAPDH (B), phospho-AMPK/total AMPK (C) and PPARα/GAPDH (E). These values were obtained by quantification of the intensity of each band of the western blot. The values are expressed as the mean ± SD from three animals in each group. ^##^p<0.01 *vs*. normal group, *p<0.05, **p<0.01 *vs*. HFD group.

To further understand the lipid-lowering effect and mechanism of wedelolactone, we applied a model of hyperlipidemia in hamsters induced by a HFD. We showed that the plasma levels of TC, TG, and LDL-C (Fig 6A–6C) were significantly decreased by treatment of wedelolactone at higher doses of 25 and 40 mg/kg. Meanwhile, TC and TG levels in the liver (Fig 7) were significantly lower in all wedelolactone-treated groups compared to the HFD group. The activation of AMPK and the up-regulation of PPARα in the liver (Fig 8) were observed in wedelolactone-treated groups in this HFD-induced hamster model, consistent with our *in vitro* HepG2 cell study (Fig 1).

**Fig 9:**
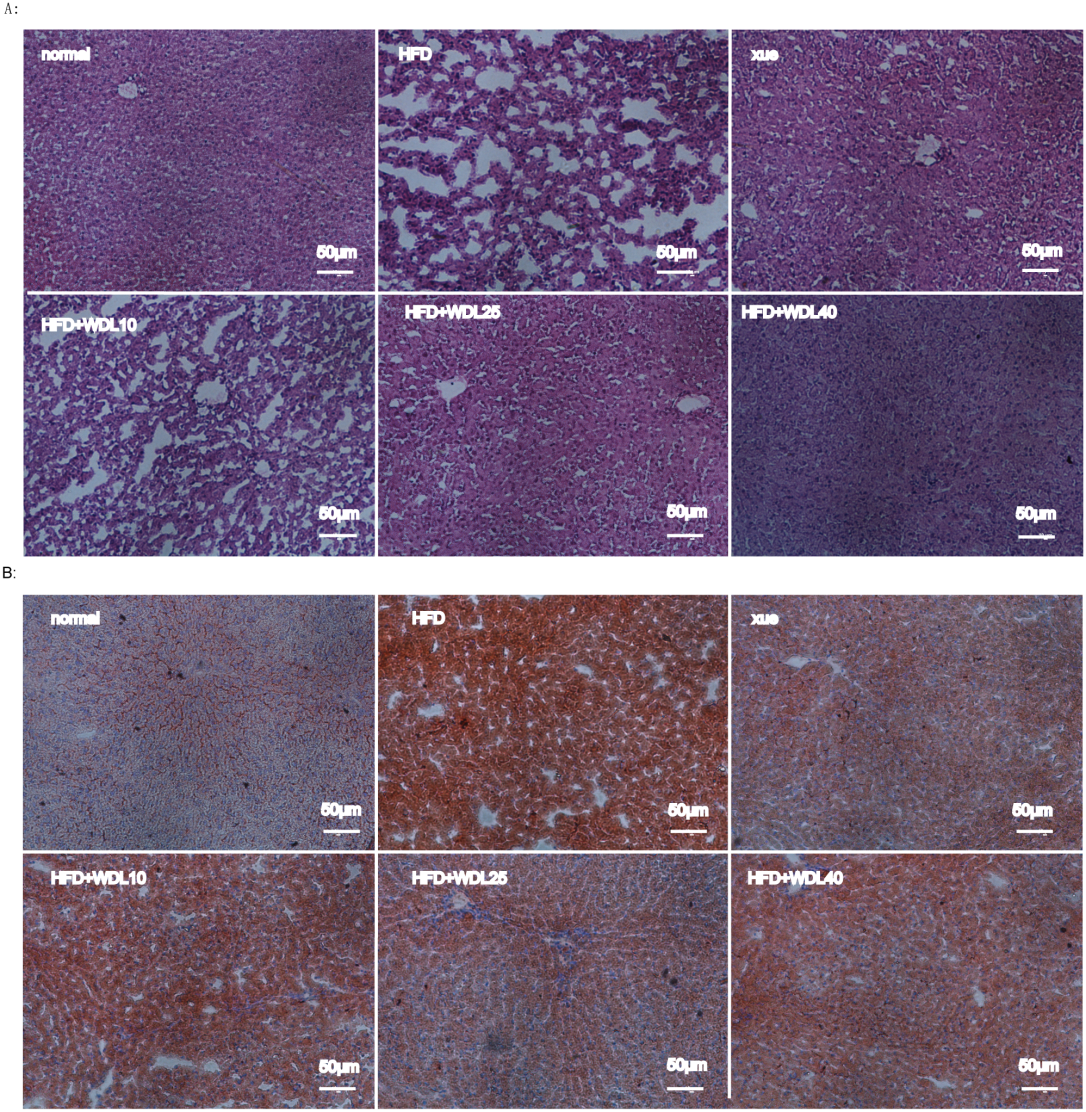
Wedelolactone alleviated liver damage and lipid accumulation. (A) Histopathologic sections of liver from Syrian hamsters with different treatments were stained with H&E. (B) Histopathologic sections of liver from hamsters in each group were stained with Oil Red O. The normal group was fed a normal diet whereas the other groups were fed a HFD. HFD + xue and HFD + WDL groups were supplemented concurrently with xuezhikang (250 mg/kg) or wedelolactone (10 mg/kg, 25 mg/kg, 40 mg/kg).

We also performed Oil Red O staining of the liver to observe hepatic lipid accumulation. It was revealed that a HFD induced significant lipid accumulation in the liver, and that wedelolactone, at three doses, inhibited lipid accumulation to be close to that of healthy animals (Fig 9B). These data were consistent with the above hepato-protective effects of wedelolactone on hepatic levels of TC and TG.

### Wedelolactone possesses anti-oxidant and hepatoprotective activities

**Fig 10:**
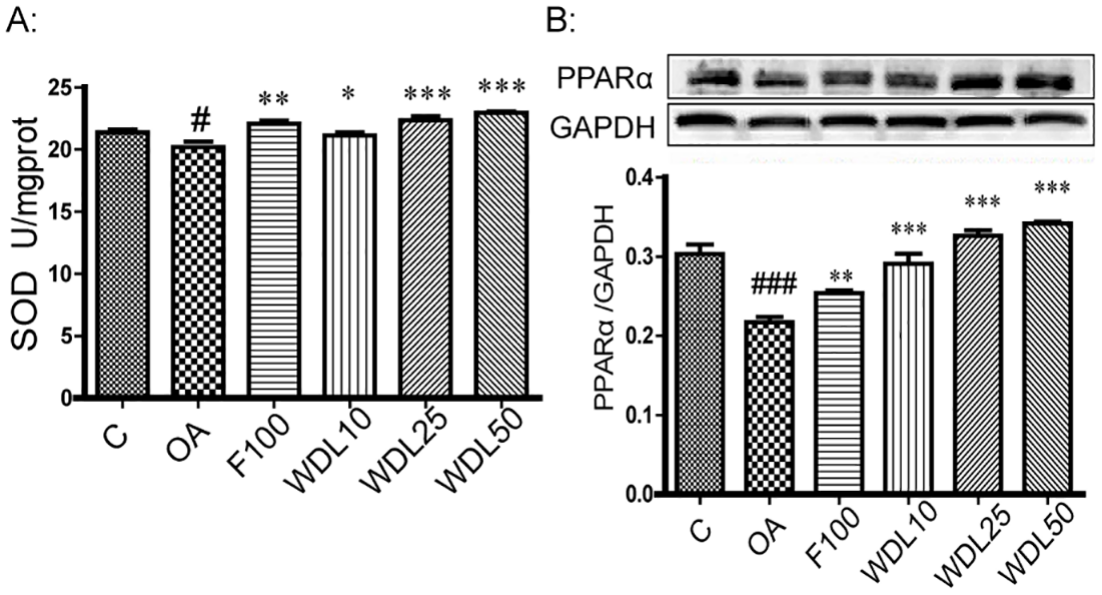
Wedelolactone increased the activities of SOD and up-regulated the expression of PPARα in OA-induced lipid peroxidized HepG2 cells. HepG2 cells were stimulated with our without OA (25 μM) and treated with or without wedelolactone at different concentrations for 24 h before collection of cell lysates for SOD detection (A) and cell proteins for western blotting determination (B). OA, F100, WDL10, WDL25 and WDL50 groups were stimulated simultaneously with OA and treated with dimethyl sulfoxide (DMSO), fenofibrate (100 μM), or wedelolactone at 10, 25 or 50 μM, respectively. The values are expressed as the mean ± SD of triplicate assays. ^#^p<0.05, ^###^p<0.001 *vs*. control group, *p<0.05, **p<0.01, ***p<0.001 *vs*. OA group.

To further illustrate the mechanism of hepatoprotective effects of wedelolactone, we evaluated whether wedelolactone had anti-oxidant activity, as the HFD model we used could induce ROS generation [17]. We demonstrated that in OA-induced lipid peroxidized HepG2 cells, wedelolactone increased the activity of SOD, accompanied by an up-regulation of PPARα (Fig 10) and possessed antiradical activity as determined by DPPH assay (Table 3). Based on the DPPH assay, the EC50 of wedelolactone was calculated as 46 μg/mL.

**Table 3:** The antioxidant activity of wedelolactone in vitro.

Groups	Concentrations(μg/mL)	Antiradical activity (%)
Control	—	0
WDL3	3	27.40±0.9498***
WDL50	50	40.78±0.8461***
WDL60	60	47.87±0.5623***
WDL80	80	58.61±0.4850***
WDL100	100	65.27±0.6370***
WDL125	125	65.63±0.6180***
WDL250	250	66.89±0.7150***
WDL500	500	66.86±0.2138***
WDL1000	1000	67.71±0.1790***

The values are expressed as the mean ±SD of triplicate assays.

***p<0.001 compared to control.

**Fig 11:**
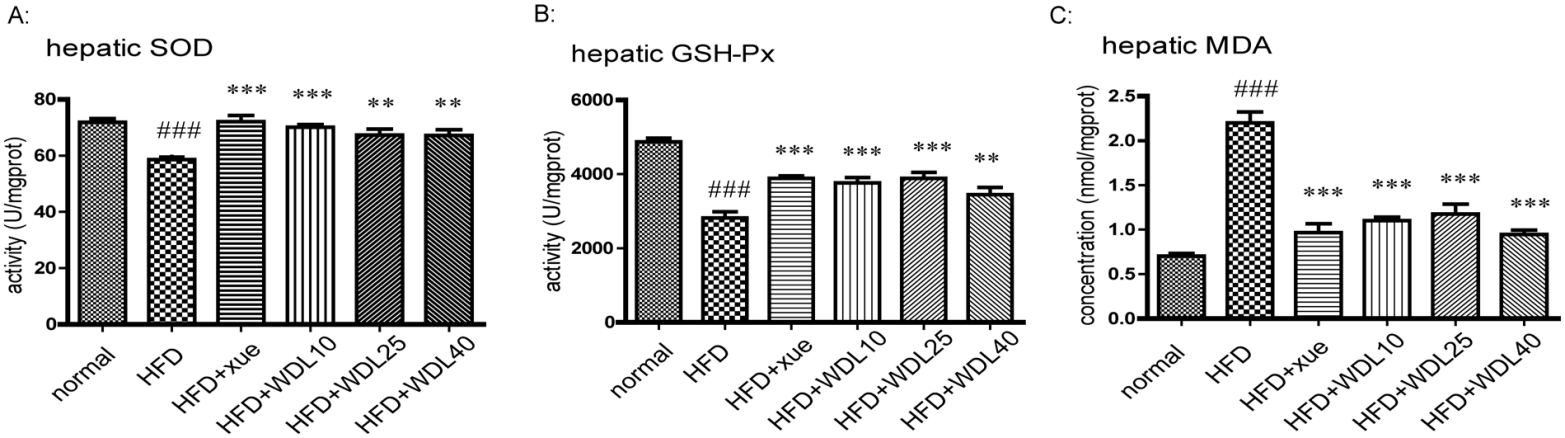
Hepatic levels of MDA (A), SOD (B) and GSH-Px (C). The normal group was fed a normal diet whereas the other groups were fed a HFD. HFD + xue and HFD + WDL groups were supplemented concurrently with xuezhikang (250 mg/kg) or wedelolactone (10 mg/kg, 25 mg/kg, 40 mg/kg). The values are shown as the mean ± SD from ten animals in each group. ^###^p<0.001 *vs*. normal group, **p<0.01, ***p<0.001 *vs*. HFD group.

Additionally, increased SOD and GSH-Px levels were observed (Fig 11A and 11B) along with a concomitant decrease in MDA (product of lipid peroxidation) (Fig 11C) and therefore decreased ALT activity (indicator of liver damage) (Fig 6D). Furthermore, the fragmentation of the hepatic cord caused by HFD was improved in wedelolactone-treated groups which were observed in the H&E stained liver sections (Fig 9A). These results suggest that wedelolactone possesses anti-oxidant and hepato-protective activities.

## Discussion

To date, studies regarding the hypolipidemic effect of wedelolactone have not been reported. Hence, we first assessed the lipid-lowering effect of wedelolactone in an acute model of hyperlipidemia induced by intraperitoneal injection of Triton WR-1339 and then in a HFD-induced model of hyperlipidemia in hamsters. Triton WR-1339 is a well-known non−ionic detergent used to induce acute hyperlipidemia in several animal models because of its ability to block TG-rich lipoprotein clearance and to increase biosynthesis of cholesterol in the liver [18]. In our acute experiment, lipid levels (TC, TG) in mice plasma were increased by Triton WR-1339 (Fig 5), identical to the data of other research groups [19,20]. Our results demonstrated that wedelolactone dramatically decreased the TG level. It has been reported that treatment with Triton WR-1339 significantly lowers LPL expression [21], implying that the lipid-reducing effect of wedelolactone may be related to enzymes such as LPL, which is responsible for clearance of TG-rich lipoproteins.

Compared to rats and mice, hamsters are considered to be the best animal model for studying lipid metabolism because metabolism patterns of lipoproteins and bile acids in hamsters are similar to that in humans [22]. The lipid profile and hepatic steatosis increased dramatically after hamsters were fed a HFD for 4 weeks. Wedelolactone improved plasma levels of TC, TG, and LDL-C as well as lipid accumulation in the liver (Figs 6, 7, and 9B). These results demonstrate that wedelolactone has admirable lipid-lowering and hepato-protective effects *in vivo*. Furthermore, we observed that the hypolipidemic effect of wedelolactone was due, at least in part, to the activation of PPARα and AMPK.

PPARα plays an important role in the regulation of lipid metabolism [23]. PPARα activation by ligands up-regulates expression of the genes involved in the transport and oxidation of fatty acids, such as LPL, CPT1, and CD36 [24–26]. LPL is a known PPARα target gene and is the rate-limiting enzyme for the hydrolysis of the TG core of circulating TG-rich lipoproteins [27]. It has been reported that the hypo- triglyceridemic action of PPARα agonists results (at least in part) from induction of the expression and activity of LPL [24,28]. Wedelolactone up-regulated PPARα both at the protein and mRNA levels in HepG2 cells (Figs 1 and 3C). With the dramatic activation of PPARα, the mRNA transcription of LPL increased accordingly (Fig 3D). Furthermore, PPARα/LPL activation demonstrated a degree of selectivity because other downstream targets of PPARα, such as CPT1 and CD36, were not significantly affected by wedelolactone (Fig 4). Consistent with the *in vitro* study, up-regulation of PPARα was also observed in wedelolactone treatment in our hamster experiment (Fig 8D–8E).

PPARα has also been reported to up-regulate SREBP-1c expression [29], which increases the synthesis of cholesterol, fatty acids, and TG by enhancing transcription of the crucial genes involved in lipogenesis, such as HMGCR, ACC, FAS, and SCD [30]. Hence, several PPARα agonists increase hepatic steatosis, which results in the development of this side effect [9,31]. Nevertheless, wedelolactone treatment improved lipid accumulation in liver (Figs 7 and 9), and it did not affect gene expression of SREBP-1c (Fig 4), most likely due to the simultaneous activation of AMPK.

AMPK is another key regulator of lipid metabolism. AMPK has a profound effect on cholesterol synthesis, fatty-acid oxidation, hepatic fatty acids, and VLDL synthesis [32,33]. In contrast to PPARα activation, AMPK phosphorylation suppresses the activity of the key proteins involved in lipogenesis, such as SREBP-1c and HMGCR [34,35], and improves liver steatosis [36]. AMPK has been proposed as a major therapeutic target for obesity and obesity-linked metabolic disorders such as hyperlipidemia [32]. Wedelolactone dose-dependently up-regulated AMPK at both the protein and mRNA levels in HepG2 cells (Figs 1 and 3A). However, ACC expression (a marker of AMPK activation and an important enzyme controlling the biosynthesis and oxidation of fatty acids [37]) remained unchanged (Fig 4) at the mRNA level in HepG2 cells. Nevertheless, our result is consistent with previous report demonstrating that ACC inhibition by AMPK can be attributed to the level of protein expression and not to the level of mRNA transcription [38]. Our hamster study demonstrated that a HFD impaired the activity of AMPK in liver (Fig 8A–8C), which was consistent with previous reports [39], and treatment of wedelolactone significantly improved it, which is consistent with the *in vitro* activation of AMPK (Fig 1).

It was reported that LDLR gene promoter activity was enhanced through the proteolytic activation of SREBP-2 and SREBP-1 [40]. However, our result showed the mRNA level of LDLR was increased markedly by wedelolactone, despite the unchanged gene expression of SREBPs, suggesting that LDLR may be regulated by other molecular mechanisms, which requires further investigation.

Wedelolactone showed beneficial anti-oxidant activity as well as lipid-lowering effects in the HFD-induced model of hyperlipidemia in hamsters. ROS are commonly generated by a HFD, and they have been observed in organs and tissues [41]. Wedelolactone demonstrated anti-oxidant effects in the DPPH assay, suggesting that it could directly eliminate ROS (Table 3). With the EC50 of 46 μg/mL, it is a stronger ROS scavenger than ascorbic acid (EC50 = 110.77 μg/mL) [42]. In addition, wedelolactone increased the SOD activity in both the *in vitro* cell study (Fig 10A) and the *in vivo* hamster study (Fig 11A). A concomitant up-regulation of PPARα in OA-induced lipid peroxidized HepG2 cells was observed (Fig 10B). In fact, the activity of SOD (one of the most important anti-oxidant enzymes) typically improves with PPARα activation because PPARα localizes to the promoter region of Cu/Zn-SOD [43], a principal type of SOD. These results suggest that wedelolactone has a direct ROS-scavenging ability and increases the activities of several antioxidant enzymes. Therefore, wedelolactone ameliorates liver damage from ROS, as evidenced by the reduced levels of ALT (Fig 6D) and improved hepatic sinusoids (Fig 9A).

In conclusion, we investigated the lipid-regulatory activities of wedelolactone. Through *in vitro* and *in vivo* studies, we demonstrated that wedelolactone possesses obvious lipid-lowering effects and its underlying mechanism is mediated by the up-regulated expression of PPARα/LPL and LDLR and the activation of AMPK. Wedelolactone was also beneficial against hepatic steatosis. Wedelolactone protected the liver from ROS damage by directly scavenging ROS and increasing the activities of several antioxidant enzymes. Therefore, wedelolactone is a promising compound in the pharmacotherapy of hyperlipidemia with the benefit of liver protection. Because the disease of lipid metabolism disorder is usually complicated by hepatic steatosis and other types of liver damage, wedelolactone has the potential to be an advantageous lipid-lowering drug.

## Supporting Information

S1 Fig Solubility curves of the mix of qPCR experiments. (TIF)

S2 Fig Agarose gel strips of the mix of qPCR experiments. (TIF)

## References

[1] Sharma N, Garg V. (2009). Antidiabetic and antioxidant potential of ethanolic extract of Butea monosperma leaves in alloxan-induced diabetic mice. Indian J Biochem Biophys 46: 99–105. 19374261

[2] Esposito K. (2002). Inflammatory Cytokine Concentrations Are Acutely Increased by Hyperglycemia in Humans: Role of Oxidative Stress. Circulation 106: 2067–72. 12379575 10.1161/01.cir.0000034509.14906.ae

[3] Xia Y, Chen J, Cao Y, Xu C, Li R, Ran Y, et al (2013)Wedelolactone exhibits anti-fibrotic effects on human hepatic stellate cell line LX-2. Eur J Pharmacol 714: 105–11. 10.1016/j.ejphar.2013.06.012 23791612

[4] Liu YQ, Zhan LB, Liu T, Cheng MC, Liu XY, Xiao HB. (2014)Inhibitory effect of Ecliptae herba extract and its component wedelolactone on pre-osteoclastic proliferation and differentiation. J Ethnopharmacol 157: 206–11. 10.1016/j.jep.2014.09.033 25267578

[5] Manvar D, Mishra M, Kumar S, Pandey VN. (2012)Identification and evaluation of anti hepatitis C virus phytochemicals from Eclipta alba. J Ethnopharmacol 144: 545–54. 10.1016/j.jep.2012.09.036 23026306 PMC3511619

[6] Kim DI, Lee SH, Choi JH, Lillehoj HS, Yu MH, Lee GS. (2008)The butanol fraction of Eclipta prostrata (Linn) effectively reduces serum lipid levels and improves antioxidant activities in CD rats. Nutr Res 28: 550–4. 10.1016/j.nutres.2008.05.003 19083459

[7] Kobori M, Yang Z, Gong D, Heissmeyer V, Zhu H, Jung YK, et al (2004)Wedelolactone suppresses LPS-induced caspase-11 expression by directly inhibiting the IKK complex. Cell Death Differ 11: 123–30. 14526390 10.1038/sj.cdd.4401325

[8] Werz O. (2007)Inhibition of 5-lipoxygenase product synthesis by natural compounds of plant origin. Planta Med 73: 1331–57. 17939102 10.1055/s-2007-990242

[9] Yan F, Wang Q, Xu C, Cao M, Zhou X, Wang T, et al (2004)Peroxisome proliferator-activated receptor alpha activation induces hepatic steatosis, suggesting an adverse effect. PLoS One 9: e99245. 10.1371/journal.pone.0099245 PMC4057124 24926685

[10] Keating GM. (2011)Fenofibrate: a review of its lipid-modifying effects in dyslipidemia and its vascular effects in type 2 diabetes mellitus. Am J Cardiovasc Drugs 11: 227–47. 10.2165/11207690-000000000-00000 21675801

[11] Tang JJ, Li JG, Qi W, Qiu WW, Li PS, Li BL, et al (2011)Inhibition of SREBP by a small molecule, betulin, improves hyperlipidemia and insulin resistance and reduces atherosclerotic plaques. Cell Metab 13: 44–56. 10.1016/j.cmet.2010.12.004 21195348

[12] Ebrahimzadeh MA, Nabavi SM, Nabavi SF, Bahramian F, Bekhradnia AR. (2010)Antioxidant and free radical scavenging activity of H. officinalis L. var. angustifolius, V. odorata, B. hyrcana and C. speciosum. Pak J Pharm Sci 23: 29–34. 20067863

[13] Schoonjans K, Peinado-Onsurbe J, Lefebvre AM, Heyman RA, Briggs M, Deeb S, Staels B, et al (1996)PPARalpha and PPARgamma activators direct a distinct tissue-specific transcriptional response via a PPRE in the lipoprotein lipase gene. EMBO J 15: 5336–48. 8895578 PMC452277

[14] Yin SN, Liu M, Jing DQ, Mu YM, Lu JM, Pan CY. (2014)Telmisartan increases lipoprotein lipase expression via peroxisome proliferator-activated receptor-alpha in HepG2 cells. Endocr Res 39: 66–72. 10.3109/07435800.2013.828741 24067162

[15] Yin J, Luo Y, Deng H, Qin S, Tang W, Zeng L, et al (2014)Hugan Qingzhi medication ameliorates hepatic steatosis by activating AMPK and PPARalpha pathways in L02 cells and HepG2 cells. J Ethnopharmacol 154: 229–39. 10.1016/j.jep.2014.04.011 24735863

[16] Dyroy E, Rost TH, Pettersen RJ, Halvorsen B, Gudbrandsen OA, Ueland T, et al (2007)Tetradecylselenoacetic acid, a PPAR ligand with antioxidant, antiinflammatory, and hypolipidemic properties. Arterioscler Thromb Vasc Biol 27: 628–34. 17185614 10.1161/01.ATV.0000255950.70774.d5

[17] Youn JY, Siu KL, Lob HE, Itani H, Harrison DG, Cai H. (2014)Role of vascular oxidative stress in obesity and metabolic syndrome. Diabetes 63: 2344–55. 10.2337/db13-0719 24550188 PMC4066332

[18] Harnafi H, Bouanani Nel H, Aziz M, Serqhini Caid H, Ghalim N, Amrani S. (2007)The hypolipidaemic activity of aqueous Erica multiflora flowers extract in Triton WR-1339 induced hyperlipidaemic rats: a comparison with fenofibrate. J Ethnopharmacol 109: 156–60. 17092671 10.1016/j.jep.2006.09.017

[19] Rony KA, Ajith TA, Nima N, Janardhanan KK. (2014)Hypolipidemic activity of Phellinus rimosus against triton WR-1339 and high cholesterol diet induced hyperlipidemic rats. Environ Toxicol Pharmacol 37: 482–92. 10.1016/j.etap.2014.01.004 24561532

[20] Kumar D, Parcha V, Maithani A, Hhulia I. (2012)Effect and evaluation of antihyperlipidemic activity guided isolated fraction from total methanol extract of Salvadora oleoides (Decne.) in Triton WR-1339 Induced hyperlipidemic rats. Pharmacogn Mag 8: 314–8. 10.4103/0973-1296.103663 24082636 PMC3785170

[21] Yao N, He RR, Zeng XH, Huang XJ, Du TL, Cui JC, et al (2014)Hypotriglyceridemic effects of apple polyphenols extract via up-regulation of lipoprotein lipase in triton WR-1339-induced mice. Chin J Integr Med 20: 31–5. 10.1007/s11655-012-1243-3 23001493

[22] Spady DK, Stange EF, Bilhartz LE, Dietschy JM. (1986)Bile acids regulate hepatic low density lipoprotein receptor activity in the hamster by altering cholesterol flux across the liver. Proc Natl Acad Sci U S A 83: 1916–20. 3456612 10.1073/pnas.83.6.1916 PMC323195

[23] Rakhshandehroo M, Sanderson LM, Matilainen M, Stienstra R, Carlberg C, de Groot PJ, et al (2007)Comprehensive analysis of PPARalpha-dependent regulation of hepatic lipid metabolism by expression profiling. PPAR Res 2007: 26839 10.1155/2007/26839 18288265 PMC2233741

[24] Schafer HL, Linz W, Falk E, Glien M, Glombik H, Korn M, et al (2012)AVE8134, a novel potent PPARalpha agonist, improves lipid profile and glucose metabolism in dyslipidemic mice and type 2 diabetic rats. Acta Pharmacol Sin 33: 82–90. 10.1038/aps.2011.165 22212431 PMC4010268

[25] Do GM, Kwon EY, Ha TY, Park YB, Kim HJ, Jeon SM, et al (2011)Tannic acid is more effective than clofibrate for the elevation of hepatic beta-oxidation and the inhibition of 3-hydroxy-3-methyl-glutaryl-CoA reductase and aortic lesion formation in apo E-deficient mice. Br J Nutr 106: 1855–63. 10.1017/S000711451100256X 21736774

[26] Peschel D, Koerting R, Nass N. (2007)Curcumin induces changes in expression of genes involved in cholesterol homeostasis. The Journal of Nutritional Biochemistry 18: 113–9. 16713233 10.1016/j.jnutbio.2006.03.007

[27] Davies BS, Beigneux AP, Fong LG, Young SG. (2012)New wrinkles in lipoprotein lipase biology. Curr Opin Lipidol 23: 35–42. 10.1097/MOL.0b013e32834d0b33 22123668 PMC3383841

[28] Staels B, Auwerx J. (1992)Perturbation of developmental gene expression in rat liver by fibric acid derivatives: lipoprotein lipase and alpha-fetoprotein as models. Development 115: 1035–43. 1280557 10.1242/dev.115.4.1035

[29] Fernandez-Alvarez A, Alvarez MS, Gonzalez R, Cucarella C, Muntane J, Casado M. (2011)Human SREBP1c expression in liver is directly regulated by peroxisome proliferator-activated receptor alpha (PPARalpha). J Biol Chem 286: 21466–77. 10.1074/jbc.M110.209973 21540177 PMC3122206

[30] Horton JD, Goldstein JL, Brown MS. (2002)SREBPs: activators of the complete program of cholesterol and fatty acid synthesis in the liver. J Clin Invest 109: 1125–31. 11994399 10.1172/JCI15593 PMC150968

[31] Rull A, Geeraert B, Aragones G, Beltran-Debon R, Rodriguez-Gallego E, Garcia-Heredia A, et al (2014)Rosiglitazone and fenofibrate exacerbate liver steatosis in a mouse model of obesity and hyperlipidemia. A transcriptomic and metabolomic study. J Proteome Res 13: 1731–43. 10.1021/pr401230s 24479691

[32] Lage R, Dieguez C, Vidal-Puig A, Lopez M. (2008)AMPK: a metabolic gauge regulating whole-body energy homeostasis. Trends Mol Med 14: 539–49. 10.1016/j.molmed.2008.09.007 18977694

[33] Slack C, Foley A, Partridge L. (2012). Activation of AMPK by the putative dietary restriction mimetic metformin is insufficient to extend lifespan in Drosophila. PLoS One 7: e47699 10.1371/journal.pone.0047699 23077661 PMC3473082

[34] Kim do Y, Yuan HD, Chung IK, Chung SH. (2009)Compound K, intestinal metabolite of ginsenoside, attenuates hepatic lipid accumulation via AMPK activation in human hepatoma cells. J Agric Food Chem 57: 1532–7. 10.1021/jf802867b 19182950

[35] Kahn BB, Alquier T, Carling D, Hardie DG. (2005)AMP-activated protein kinase: ancient energy gauge provides clues to modern understanding of metabolism. Cell metabolism 1: 15–25. 16054041 10.1016/j.cmet.2004.12.003

[36] Li Y, Xu S, Mihaylova MM, Zheng B, Hou X, Jiang B, et al (2011)AMPK phosphorylates and inhibits SREBP activity to attenuate hepatic steatosis and atherosclerosis in diet-induced insulin-resistant mice. Cell Metab 13: 376–88. 10.1016/j.cmet.2011.03.009 21459323 PMC3086578

[37] Guo P, Kai Q, Gao J, Lian ZQ, Wu CM, WU CA, et al (2010)Cordycepin Prevents Hyperlipidemia in Hamsters Fed a High-Fat Diet via Activation of AMP-Activated Protein Kinase. Journal of Pharmacological Sciences 113: 395–403. 20724804 10.1254/jphs.10041fp

[38] Zhang X, Wu C, Wu H, Sheng L, Su Y, Zhang X, et al (2013)Anti-hyperlipidemic effects and potential mechanisms of action of the caffeoylquinic acid-rich Pandanus tectorius fruit extract in hamsters fed a high fat-diet. PLoS One 8: e61922 10.1371/journal.pone.0061922 23613974 PMC3628350

[39] Barroso E, Rodriguez-Calvo R, Serrano-Marco L, Astudillo AM, Balsinde J, Palomer X, et al (2011)The PPARbeta/delta activator GW501516 prevents the down-regulation of AMPK caused by a high-fat diet in liver and amplifies the PGC-1alpha-Lipin 1-PPARalpha pathway leading to increased fatty acid oxidation. Endocrinology 152: 1848–59. 10.1210/en.2010-1468 21363937

[40] Yashiro T, Nanmoku M, Shimizu M, Inoue J, Sato R. (2012)Resveratrol increases the expression and activity of the low density lipoprotein receptor in hepatocytes by the proteolytic activation of the sterol regulatory element-binding proteins. Atherosclerosis 220: 369–74. 10.1016/j.atherosclerosis.2011.11.006 22153697

[41] Lee W, Eom DW, Jung Y, Yamabe N, Lee S, Jeon Y, et al (2012)Dendrobium moniliforme attenuates high-fat diet-induced renal damage in mice through the regulation of lipid-induced oxidative stress. Am J Chin Med 40: 1217–28. 10.1142/S0192415X12500905 23227793

[42] Ricci D, Fraternale D, Giamperi L, Bucchini A, Epifano F, Burini G, et al (2005)Chemical composition, antimicrobial and antioxidant activity of the essential oil of Teucrium marum (Lamiaceae). J Ethnopharmacol 98: 195–200. 15763383 10.1016/j.jep.2005.01.022

[43] Hou X, Shen YH, Li C, Wang F, Zhang C, Bu P, et al (2010)PPARalpha agonist fenofibrate protects the kidney from hypertensive injury in spontaneously hypertensive rats via inhibition of oxidative stress and MAPK activity. Biochem Biophys Res Commun 394: 653–9. 10.1016/j.bbrc.2010.03.043 20226762

