# Supplementary figures and images for: Wedelolactone Regulates Lipid Metabolism and Improves Hepatic Steatosis Partly by AMPK Activation and Up-Regulation of Expression of PPARα/LPL and LDLR
Source: PLoS One. 2015 Jul 13;10(7):e0132720. doi: 10.1371/journal.pone.0132720 (PMC4500417; doi:10.1371/journal.pone.0132720)

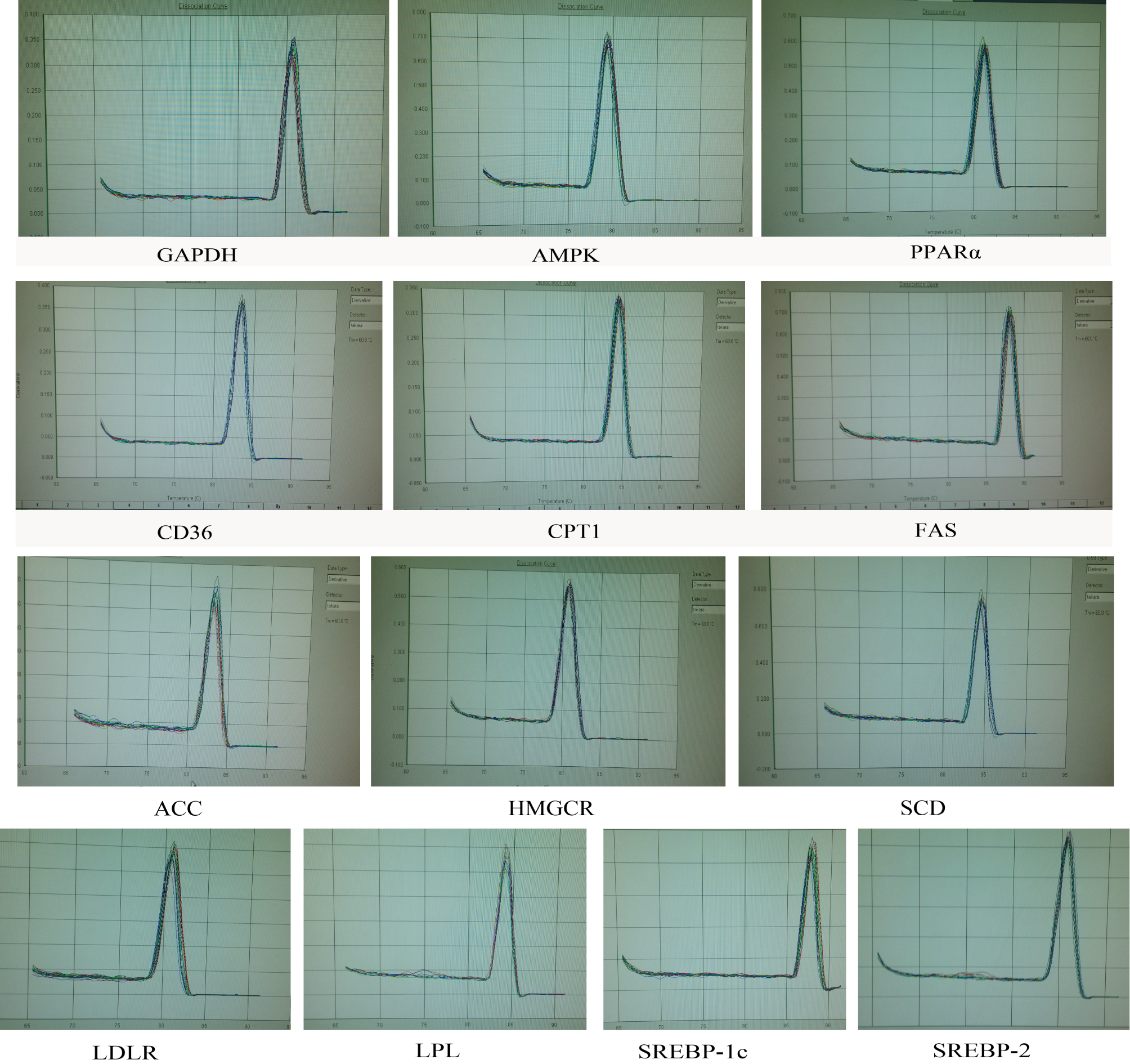

Supplement: S1 Fig — (TIF) [file pone.0132720.s001.tif]

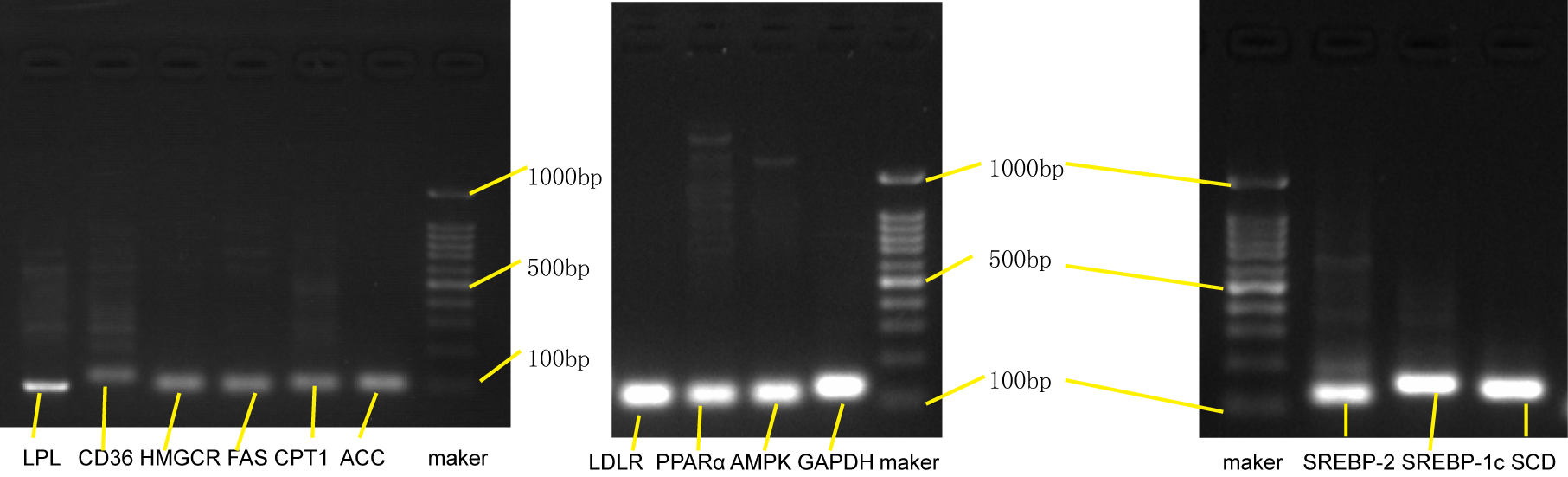

Supplement: S2 Fig — (TIF) [file pone.0132720.s002.tif]
